# Bak and Bax are crucial for Gbp2-mediated pyroptosis during *Vibrio* and *Salmonella* infections

**DOI:** 10.71150/jm.2508004

**Published:** 2025-09-30

**Authors:** Yongyang Luo, Jeehyeon Bae

**Affiliations:** 1 Department of Life Science, Chung-Ang University, Seoul 06974, Republic of Korea; 2 School of Pharmacy, Chung-Ang University, Seoul 06974, Republic of Korea

**Keywords:** pyroptosis, *Vibrio*, *Salmonella*, guanylate binding protein, Bak, Bax

## Abstract

Pyroptosis a lytic form of programmed cell death, is a crucial host defense mechanism against bacterial pathogens. While caspase-mediated pathways are central to pyroptosis, the involvement of apoptotic regulators such as Bak, Bax, and MCL-1 in bacterial infection-induced pyroptosis remains unclear. Here, we investigated how these BCL-2 family proteins modulate pyroptosis induced by *Vibrio vulnificus* and *Salmonella enterica* serovar Typhimurium in murine cells. In mouse embryonic fibroblasts (MEFs), both pathogens strongly induced Gbp2 expression and activated caspase‑11, whereas activation of caspase‑1 occurred only in macrophages, indicating engagement of both non-canonical and canonical pyroptosis pathways. Importantly, *Bak*^-/-^ and *Bax*^-/-^ MEFs exhibited significantly reduced Gbp2 upregulation and caspase-11 activation-an effect most pronounced in Bak-deficient cells leading to attenuated pyroptotic cell death. These data suggest that pro-apoptotic proteins, Bak and Bax, act as positive regulators that amplify the Gbp2-caspase-11 axis. Conversely, overexpression of the anti-apoptotic protein MCL‑1 had no significant impact on Gbp2 expression, caspase activation, membrane integrity, or LDH release, indicating that pyroptosis proceeds independently of MCL‑1 regulation. Collectively, our findings uncover a novel role for Bak and Bax in promoting Gbp2-driven pyroptosis during Gram-negative bacterial infections, while MCL‑1 does not impede this process. This work expands our understanding of the crosstalk between apoptotic and pyroptotic pathways in innate immune responses.

## Introduction

Pyroptosis is a form of programmed lytic cell death triggered by bacterial infection and serves as a critical innate immune defense mechanism. Distinguished from apoptosis and necrosis by its rapid cell swelling, membrane rupture, and secretion of pro-inflammatory cytokines, pyroptosis plays a pivotal role in pathogen clearance (Wei et al., 2022). Dysregulated pyroptosis has been implicated in a variety of diseases, including infectious, autoimmune, and neurodegenerative disorders (Liu et al., 2024; Yu et al., 2021). However, studies on pyroptosis caused by bacterial infections especially for *Vibrio* are very limited.

Pyroptotic pathways are primarily mediated by inflammatory caspases, caspase-1 and caspase-11 in mice (Song et al., 2022). The canonical pyroptosis pathway is triggered by the recognition of pathogen-associated molecular patterns (PAMPs) or danger-associated molecular patterns (DAMPs) by pattern-recognition receptors (PRRs) such as NLRP3 (NOD-like receptor family, pyrin domain-containing 3), forming a multi-protein complex called the inflammasome (Barnett et al., 2023). The NLRP3 inflammasome recruits and activates procaspase-1, leading to its cleavage into active caspase-1. Active caspase-1 then cleaves the pro-forms of interleukin-1β (IL-1β) and IL-18 into their mature, bioactive forms, which are secreted and initiate a robust inflammatory response (Ye et al., 2023). Caspase-1 can also directly cleave gasdermin D (GSDMD), generating a GSDMD-N-terminal fragment (Stoess et al., 2023). This fragment oligomerizes and inserts into the cell membrane, forming pores that cause cell swelling and lysis, ultimately resulting in pyroptosis (Ding et al., 2016; Schroder and Tschopp, 2010).

The non-canonical pathway is triggered by caspase-11 sensing cytosolic lipopolysaccharide (LPS) in mice (caspase‑4/5 in humans) (Wright et al., 2022). Once activated, caspase-11 also cleaves GSDMD, leading to pyroptosis (Kayagaki et al., 2015). Moreover, activated caspase-11 can indirectly activate caspase-1 through an NLRP3 inflammasome-dependent or -independent manner, further amplifying the inflammatory response (Downs et al., 2020; Kayagaki et al., 2015).

Gbp2 belongs to the guanylate-binding protein family, which is known for its function in host cell immunity and antimicrobial activity (Feng et al., 2022). Gbp2 is an interferon (IFN)-inducible GTPase known to be pivotal in innate immunity against a wide array of bacterial, viral, and protozoan pathogens (Kirkby et al., 2023). Gbp2 contributes to the inflammasome activation in the pyroptosis pathway (Dickinson et al., 2023; Pilla et al., 2014). Upon infection, Gbp2 is recruited to pathogen-containing vacuoles or cytosolic bacteria, facilitating vacuolar lysis and release of microbial ligands that trigger inflammasome assembly (Kirkby et al., 2023; Kutsch and Coers, 2021).

The BCL-2 family is an evolutionally conserved group of proteins that play essential roles in regulating apoptotic cell death and cell survival (Hatok and Racay, 2016; Qian et al., 2022). This family comprises of both anti-apoptotic and pro-apoptotic subfamilies (Qian et al., 2022). Among the pro-apoptotic members, BAK and BAX serve as key mediators of mitochondrial-dependent apoptosis (Czabotar and García-Sáez, 2023; Kaloni et al., 2023). In response to apoptotic stimuli, BAK and BAX undergo conformational changes and oligomerize at the mitochondrial outer membrane, leading to mitochondrial outer membrane permeabilization (MOMP). This process facilitates the release of cytochrome c into the cytosol and the subsequent activation of caspases, ultimately resulting in apoptosis (Cosentino and García-Sáez, 2017; Peña-Blanco and García-Sáez, 2018; Wolf et al., 2022). In contrast, anti-apoptotic members such as MCL-1 prevent apoptosis by binding to and inhibiting BAK and BAX, thereby preserving mitochondrial membrane integrity (Vogler et al., 2025).

Our previous study demonstrated that GBP2 can induce caspase-dependent apoptosis in both chronic and acute myeloid leukemia cells by directly interacting with MCL-1 (Luo et al., 2021). This interaction disrupts the MCL-1–BAK complex, releasing BAK to initiate MOMP and apoptosis (Luo et al., 2021). Furthermore, GBP2 was shown to upregulate BAK expression through the PI3K/AKT signaling pathway (Luo et al., 2021). Given the dual functional role of GBP2 in both pyroptosis and apoptosis, and its regulatory interactions with MCL-1 and BAK, this study aimed to investigate whether BCL-2 family proteins also modulate pyroptosis in response to Gram-negative bacterial infections.

For this study, we selected *Vibrio vulnificus* and *Salmonella* Typhimurium based on their clinical relevance and contrasting infection dynamics. *V. vulnificus* is known to cause rapid and often fatal septicemia, particularly in immunocompromised individuals, making it an ideal model for investigating acute pyroptotic responses to high-burden bacterial infections (Jones and Oliver, 2009). In contrast, *S.* Typhimurium is a well-characterized intracellular pathogen associated with gastroenteritis and exhibits slower intracellular replication and employs immune evasion strategies that result in delayed inflammasome activation (Fàbrega and Vila, 2013). This contrast allowed us to examine how differences in pathogen lifestyle and host-pathogen interactions influence the kinetics and pathways of pyroptotic responses.

## Materials and Methods

### Mammalian cell culture

Mouse macrophage Raw 264.7 cells (Korean cell line bank, Korea) were cultured in Dulbecco’s modified Eagle’s medium (DMEM) (Caisson, USA) supplemented with 10% fetal bovine serum (Caisson) and 1% penicillin-streptomycin (Caisson). Mouse embryo fibroblasts (MEF) cells of *Bak*^-/-^, *Bax*^-/-^, and *Bak*^-/-^*Bax*^-/-^ were gifts from Dr. CB Thompson (University of Pennsylvania, USA). MEF cells were cultured in DMEM supplemented with 1 μM asparagine (Sigma-Aldrich, USA) and 50 μM 2-mercaptoethanol (Sigma-Aldrich). Cells were grown in an incubator at 37°C with 5% CO_2_.

### Bacterial culture and infection

*Vibrio vulnificus* M06-24/0 or *Salmonella enterica* serovar Typhimurium ATCC 14028 were gifts from Dr. Kangseok Lee (Chung-Ang University, Korea). Bacteria were cultured aerobically at 37°C in Luria-Bertani broth (Sigma Aldrich) for around 1–2 h until the OD_600_ reached to 0.7. Cells were harvested by centrifugation at 5,000 rpm for 5 min, and bacteria pellets were resuspended in DMEM media for the infection. Raw 264.7 cells or MEF cells were incubated overnight and washed with PBS followed with bacterial infection for 30 min (*V. vulnificus* M06-24/O) at a multiplicity of infection (MOI) of 20, or 1 h (*S.* Typhimurium ATCC 14028) at an MOI of 100. After infection, cells were washed with PBS and incubated with DMEM containing 50 mg/ml gentamicin (Gibco, USA) for indicated time.

### Plasmid constructions

The Flag-tagged MCL-1 WT expression plasmid was constructed as previously described (Luo et al., 2021).

### Cell cytotoxicity-LDH assay

Cells (1 × 10^4^) were seeded into 96-well plate and incubated overnight followed with the infection for indicated time. 50 μl of the culture supernatant from each well was transferred to a new 96-well plate after infection for the LDH detection using CytoTox 96^®^ Non-Radioactive Cytotoxicity Assay (Promega, USA) according to the manufacturer’s instructions.

### Propidium iodide (PI) uptake

PI uptake was measured to assess cell membrane integrity following bacterial infection. Cells were seeded in 96-well plates at a density of 1 × 10^4^ cells/well and incubated overnight at 37°C with 5% CO₂ to reach 70–80% confluence. Subsequently, the cells were infected with bacteria at the desired MOI and incubated for the specified time period. At indicated time, 10 μM of PI was added to each well, and the plates were incubated in the dark at room temperature for 15 min. Fluorescence intensity was then measured using a microplate reader with an excitation wavelength of 535 nm and an emission wavelength of 617 nm. Relative PI uptake was calculated to evaluate the extent of cell membrane damage caused by bacteria infections.

### Confocal microscopic analysis of PI staining

PI staining was performed to visualize cell death by confocal microscopy. Cells grown on coverslips were infected for indicated incubation period. Subsequently, 10 μM of PI was added to the cells, and they were incubated in the dark at room temperature for 15 min. Following incubation, the cells were washed with PBS to eliminate excess PI. The coverslips were then mounted onto glass slides with Fluoroshield^TM^ with DAPI mounting solution (ImmunoBioScience Crop, USA). Images were captured using a Zeiss LSM 800 confocal laser scanning microscope (Carl Zeiss, Germany) with an excitation wavelength of 535 nm and an emission wavelength of 617 nm.

### Cell viability assay

Cell viability after infection was determined using the cell counting method. Cells were harvested using trypsin-EDTA solution and then resuspended in fresh medium. A 10 μl cell suspension was mixed with 10 μl of trypan blue solution, and 10 μl of the mixture was loaded onto a hemocytometer. Under an optical microscope (Leica, Germany), cells were counted in four large squares of the hemocytometer. Viable cells, which excluded trypan blue and appeared clear, were distinguished from non-viable cells, which took up the dye and appeared blue. Cell viability was calculated as the percentage of viable cells out of the total cell count.

### Immunoblot analysis

Cells (1 × 10^6^) were transfected with indicated plasmids for 24 h with or without bacterial infection for indicated time, the cell lysates were prepared and subjected to SDS-PAGE for immunoblotting with respective antibodies. The protein signal on membranes were detected using an Amersham Imager 600 (GE Healthcare Life Sciences, UK). The rabbit anti-GBP2 (11854-1-AP) was obtained from Proteintech (Rosemont, USA). The rabbit anti-MCL-1 (sc-819), mouse anti-β-Actin (sc-47778) and mouse anti-caspase-1 (sc-56036) antibodies were purchased from Santa Cruz Biotechnology (USA). The mouse anti-Caspase-11 (14340) and rabbit anti-Gasdermin D (97558) was from Cell Signaling Technology (USA).

### RNA extraction and real-time polymerase chain reaction (PCR) analysis

Isolation of total RNA and real-time reverse-transcription PCR were carried out as described (Won et al., 2019). The nucleotide sequences of primers used for real-time PCR were as follows: MCL-1-F (5´-TGCTTCGGAAACTGGACATCA) and MCL-1-R (5´-TAGCCACAAAGGCACCAAAAG); GAPDH-F (5´-AGGGGCCATCCACAGTCTT) and GAPDH-R (5´-AGCCAAAAGGGTCATCATCTCT).

### Statistical analysis

Statistical analyses were performed using one-way ANOVA followed by Tukey’s multiple comparisons test in GraphPad Prism (GraphPad Software, USA). Different letters indicate statistically significant differences among groups (*p* < 0.05).

## Results

### Bak and Bax modulate Gbp2 expression and caspase-11 activation in pyroptosis induced by *Vibrio* and *Salmonella* infections

LPS, a component of the outer membrane of Gram-negative bacteria, is recognized by GBP proteins, which subsequently activate the non-canonical inflammasome pathway mediated by caspase11 in mice (or caspase 4/5 in humans), ultimately leading to pyroptotic cell death (Santos et al., 2018; Wandel et al., 2020). Based on this mechanism, we investigated how *Vibrio vulnificus* M06-24/0 or *Salmonella* Typhimurium 14028 modulate pyroptosis, with a particular focus on Gbp2 expression and the roles of the pro-apoptotic proteins Bak and Bax.

**Fig. 1.**
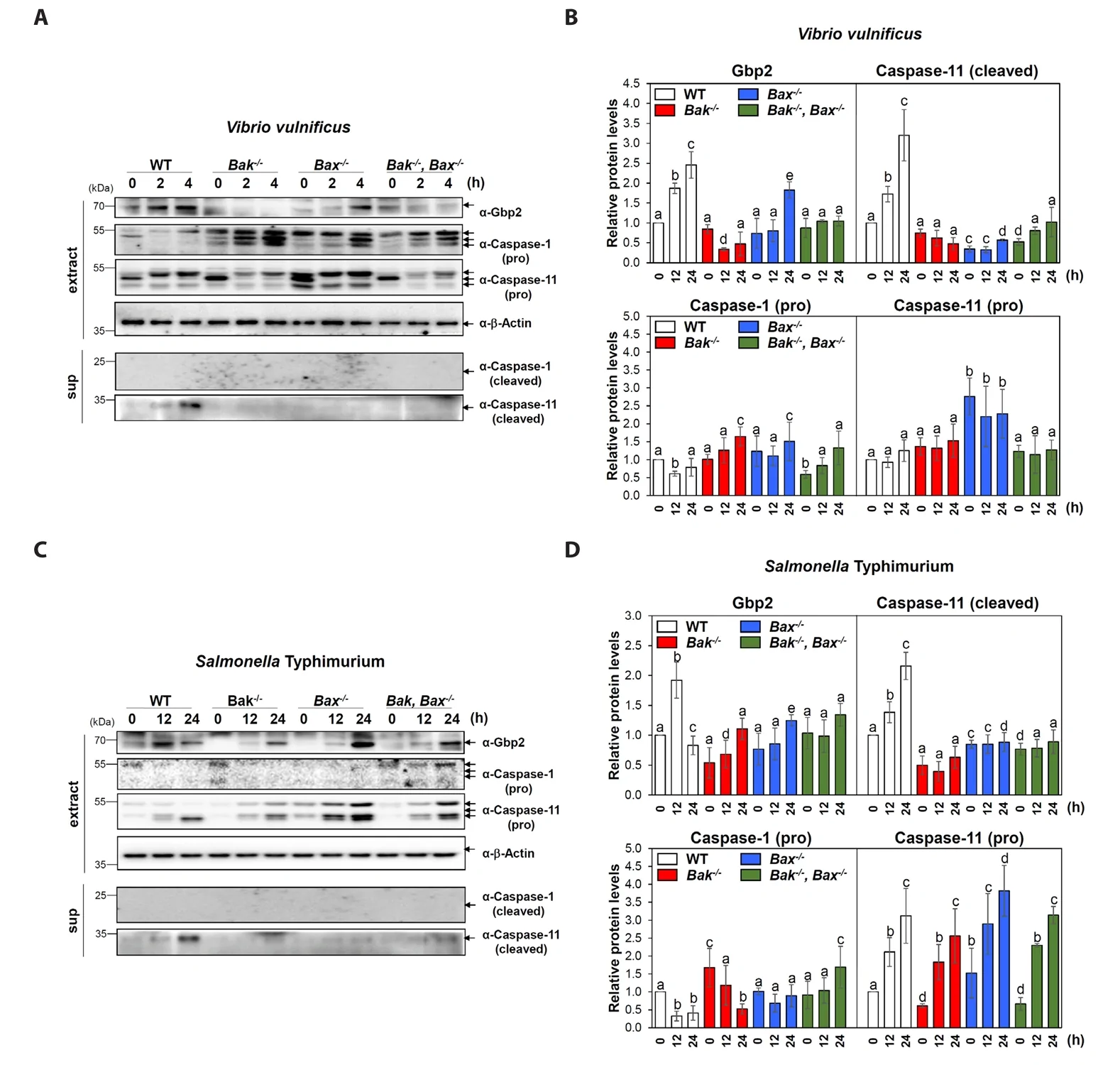
Involvement of Bak or Bax in Gbp2 upregulation and caspase-11 activation following bacterial infections. (A, C) Western blot analysis was performed to examine the expression levels of Gbp2 and the activation (cleavage) of caspase-1 and caspase-11 in wild-type (WT), *Bak*^-/-^, *Bax*^-/-^, and *Bak*^-/-^*Bax*^-/-^ MEF cells following infection with *V. vulnificus* M06-24/O at a multiplicity of infection (MOI) of 20 for 30 min followed by incubation in DMEM containing 50 µg/ml gentamicin for 0–4 h (A) or *S.* Typhimurium ATCC 14028 at an MOI of 100 for 1 h followed by incubation in DMEM containing 50 µg/ml gentamicin for 0–24 h (C). Aliquots of cell extract (extract) and supernatant (sup) were subjected to immunoblot analysis. Representative blots are shown. (B, D) Protein band intensities from western blot analyses were quantified and presented as Mean ± SEM from three independent experiments. Cleaved caspase-1 was not detectable by immunoblotting under the tested conditions. Statistically significant differences between groups were indicated by different letters (*p* < 0.05).

MEF cells with different genotypes-wild-type (WT), Bak knockout (*Bak*^-/-^), Bax knockout (*Bax*^-/-^), and both Bak/Bax double knockout (*Bak*^-/-^*Bax*^-/-^) were infected with *V. vulnificus* M06-24/0. At 2 and 4 h post-infection, WT cells showed a marked increase in Gbp2 expression and activation (cleavage) of caspase-11, while caspase-1 remained inactive (Fig. 1A and 1B). In contrast, Gbp2 induction and caspase-11 activation were significantly impaired in *Bak*^-/-^ and *Bak*^-/-^*Bax*^-/-^ MEFs, with a milder reduction observed in *Bax*^-/-^ cells (Fig. 1A and 1B). These findings indicate that *V. vulnificus* induces non-canonical pyroptosis in MEFs and that both Bak and Bax, particularly Bak, contribute to the effective activation of the Gbp2–caspase-11 axis.

Infection with *S.* Typhimurium 14028 also led to increased Gbp2 expression and caspase-11 activation in WT MEFs, but with delayed kinetics-evident at 12 and 24 h post-infection (Fig. 1C and 1D). In *Bak*^-/-^, *Bax*^-/-^, and *Bak*^-/-^*Bax*^-/-^ cells, Gbp2 upregulation was still observed, although it was delayed until 24 h and was stronger in *Bax*^-/-^ cells compared to *Bak*^-/-^ and double knockout cells (Fig. 1C and 1D). Caspase-11 activation was also markedly attenuated in all knockout cells compared to WT, while caspase-1 activation remained undetectable, further confirming non-canonical pyroptosis in this context. These results suggest that although Bak and Bax are not absolutely required for pyroptosis, they play important roles in facilitating timely and robust activation of Gbp2 and caspase-11 during *Salmonella* infection.

### Pyroptosis induced by *Vibrio* and *Salmonella* infections is dependent on Bak and Bax

**Fig. 2.**
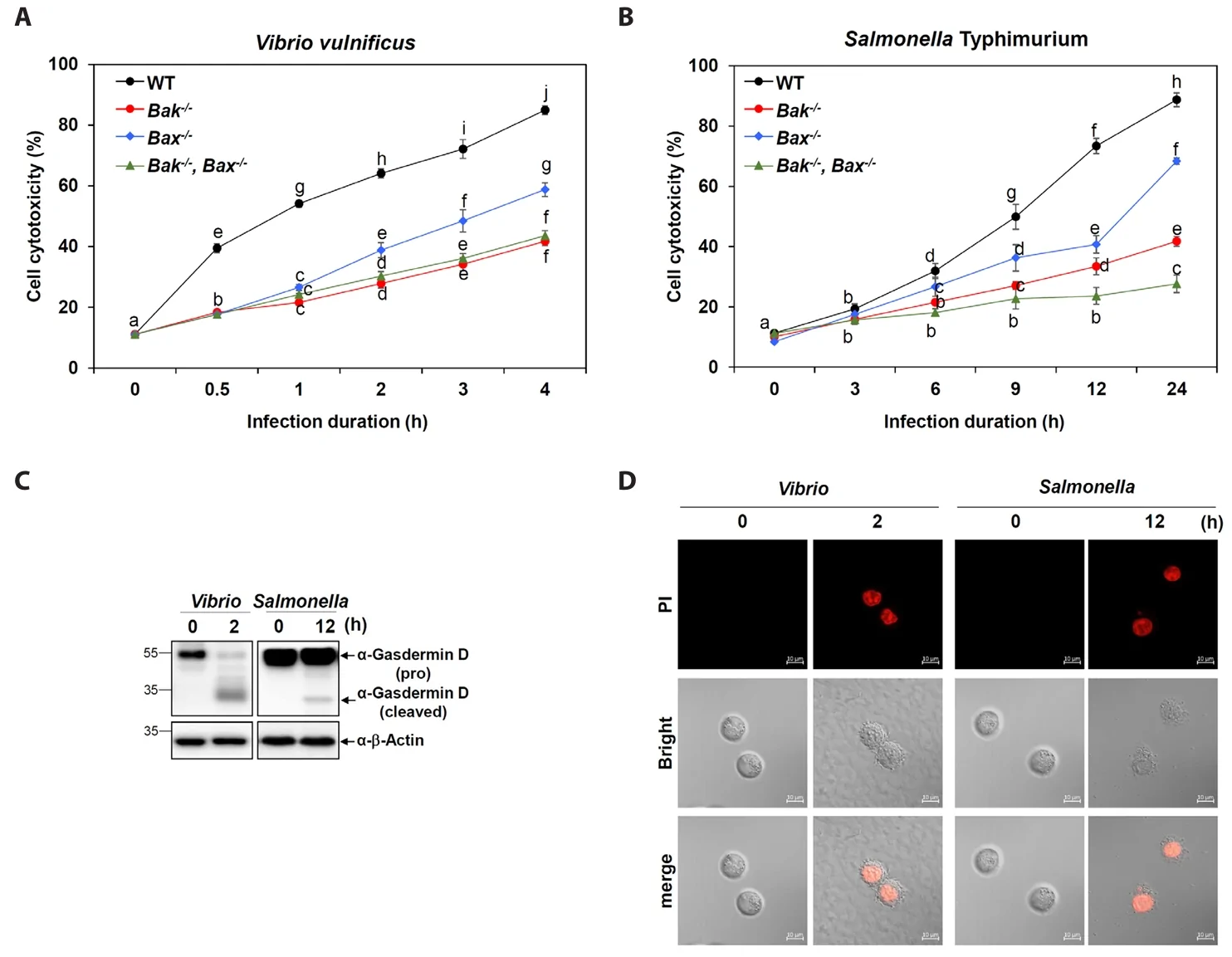
Crucial roles of Bak and Bax in *Vibrio*- and *Salmonella*-induced pyroptosis. (A, B) Cell cytotoxicity was assessed using an LDH release assay in WT and *Bak*^-/-^, *Bax*^-/-^, and *Bak*^-/-^*Bax*^-/-^ MEF cells after infection with *V. vulnificus* M06-24/O (A) for 30 min at an MOI of 20 or *S.* Typhimurium ATCC 14028 for 1 h at an MOI of 100 (B) followed by incubation in DMEM containing 50 mg/ml gentamicin for the indicated time points. Results are from three independent experiments performed in triplicates. Different letters denote statistically significant differences (*p* < 0.05). The Gasdermin D (GSDMD) cleavage (C) and membrane rupture (D) were detected in MEF WT cells with or without infection. MEF cells were infected with *V. vulnificus* M06-24/O (*Vibrio*) at a MOI of 20 for 30 min, or with *S.* Typhimurium ATCC 14028 (*Salmonella*) at a MOI of 100 for 1 h. After infection, cells were washed with PBS and incubated with DMEM containing 50 mg/ml gentamicin for the indicated times. (C) Cells lysates were collected and subjected to SDS-PAGE, followed by immunoblotting with an anti-GSDMD antibody to detect cleaved and pro-form GSDMD. β-Actin was used as a loading control. (D) Following infection as described above, MEF WT cells were incubated with 10 µM propidium iodide (PI) for 15 min at room temperature in the dark. Bright-field images were acquired to evaluate cell morphology, and merged images confirmed co-localization of PI signals with infected cells, indicating membrane rupture.

To determine the functional relevance of Bak and Bax in bacterial infection-induced cell death, we measured cytotoxicity in MEFs infected with *V. vulnificus* M06-24/0. WT cells exhibited a time-dependent increase in cytotoxicity (Fig. 2A). In contrast, *Bak*^-/-^ and *Bak*^-/-^*Bax*^-/-^ cells showed significantly reduced cytotoxicity, with *Bax*^-/-^ cells showing a moderate reduction (Fig. 2A). This pattern closely matched the changes observed in Gbp2 expression and caspase-11 activation. A similar trend was observed upon *Salmonella* infection, where Bak deficiency had a more pronounced impact on cell death than Bax deficiency (Fig. 2B).

To investigate pyroptotic responses during *Vibrio* and *Salmonella* infections, we examined GSDMD processing–a hallmark of pyroptosis– and evaluated propidium iodide (PI) uptake as an indicator of membrane permeability associated with pyroptotic cell death. In wild-type MEF cells infected with *Vibrio* or *Salmonella*, the cleaved, active form of GSDMD was detected at 2 and 12 h post-infection, respectively, accompanied by a corresponding decrease in full-length pro-GSDMD, as shown by western blot analysis (Fig. 2C). In parallel, strong red PI-positive signals were observed via fluorescence confocal microscopy at matching time points (2 h for *Vibrio* and 12 h for *Salmonella*), indicating loss of membrane integrity (Fig. 2D). Together, these data confirm that both *Vibrio* and *Salmonella* infections induce pyroptosis in host cells, albeit with distinct kinetics. Furthermore, both Bak and Bax contribute to optimal pyroptotic responses in this context, with Bak playing a more dominant role.

### *V. vulnificus* and *S.* Typhimurium infections upregulate Gbp2 and activate caspase1 and 11 in macrophage

To further explore the response in immune cells, we assessed the effects of *V. vulnificus* and *S.* Typhimurium infections in Raw 264.7 macrophages. Infection conditions were optimized by monitoring cell viability over time. A marked decrease in viability was observed starting at 30 min post-infection for *Vibrio* and at 3 h for *Salmonella* (Fig. S1A and S1B), guiding the selection of appropriate time points (2–4 h for *Vibrio*; 12–24 h for *Salmonella*) for further experiments.

**Fig. 3.**
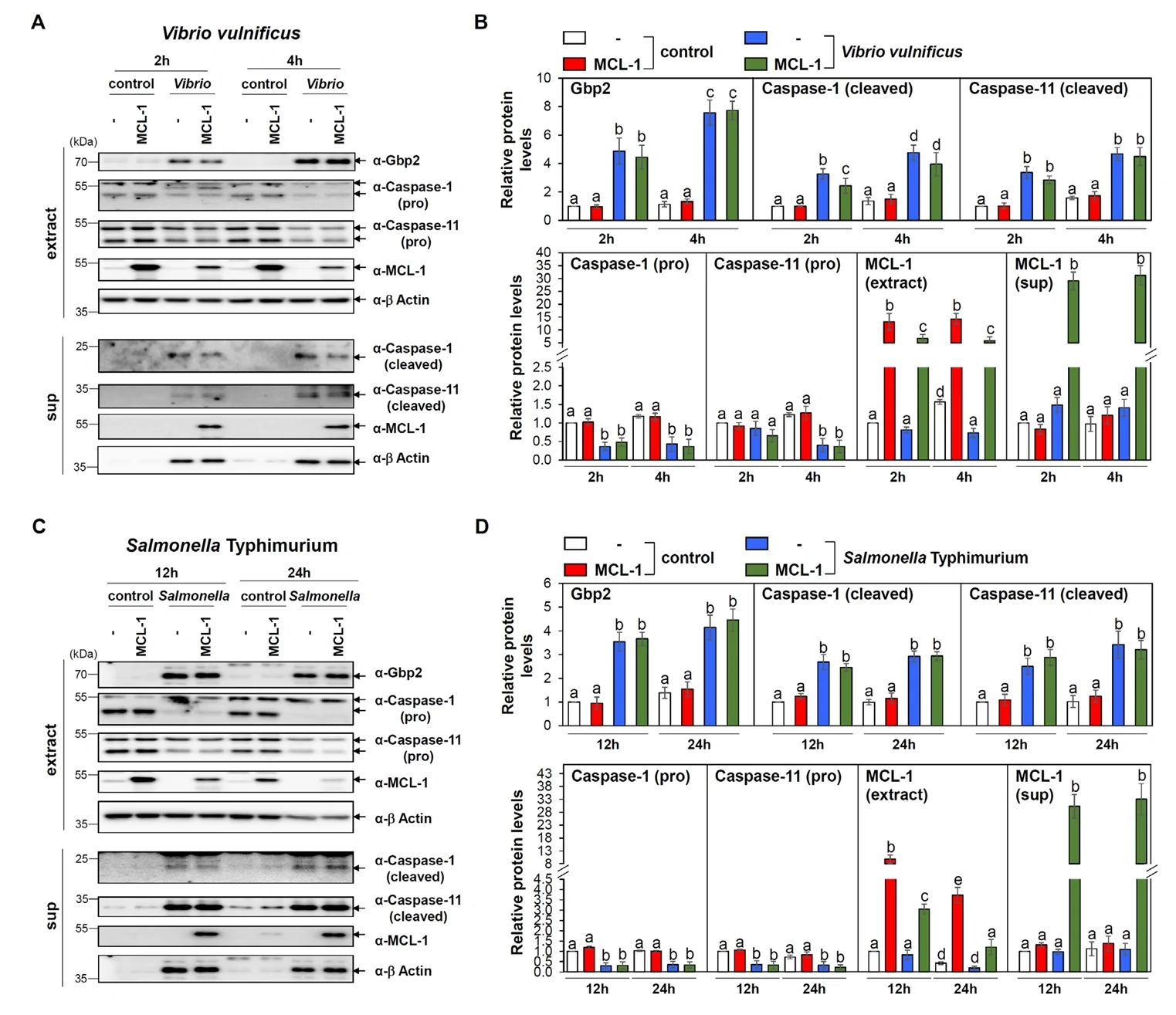
Insignificant effects of MCL-1 overexpression in Gbp2, caspase-1 and 11 and activation following *Vibrio* and *Salmonella* infections. (A) Western blot analysis was conducted to assess the expression levels of Gbp2 and cleaved caspase-1 and -11 in Raw 264.7 cells transfected with control (-) or MCL-1 expression vectors (MCL-1) and infected with *V. vulnificus* M06-24/O at an MOI of 20 for 30 min, followed by incubation in DMEM containing 50 µg/ml gentamicin for 2 or 4 h. Cleaved caspase-1 and -11 were also detected in culture supernatants. β-Actin was used as a loading control. Aliquots of cell extract (extract) and supernatant (sup) were subjected to immunoblot analysis. Representative blots are shown. (B) Protein band intensities from western blot analyses were quantified. Data presented as mean ± SEM from three independent experiments. Statistically significant differences between groups are indicated by different letters (*p* < 0.05). (C) Analogous analyses were performed following *S.* Typhimurium ATCC 14028 infection at a MOI of 100 for 1 h, followed by incubation in DMEM containing 50 µg/ml gentamicin for 12 or 24 h. Representative blots are shown. (D) Protein band intensities from western blot analyses were quantified. Data presented as mean ± SEM from three independent experiments. Statistically significant differences between groups are indicated by different letters (*p* < 0.05).

Both infections resulted in a robust upregulation of Gbp2, consistent with our observations in MEFs (Fig. 3). In addition, both caspase-1 and caspase-11 were activated in infected Raw 264.7 cells (Fig. 3), indicating that both canonical (caspase-1-dependent) and non-canonical (caspase-11-dependent) pyroptosis pathways were triggered.

Given our prior findings that GBP2 induces apoptosis via MCL-1 interaction (Luo et al., 2021), we investigated whether MCL-1 also modulates pyroptosis under these conditions. Raw 264.7 cells were transfected with either empty vector or a Flag-MCL-1 expression plasmid, followed by bacterial infections. Immunoblot analysis revealed that overexpression of MCL-1 did not significantly alter Gbp2 expression or the activation of caspase-11 in response to either *Vibrio* or *Salmonella* infection, while marginal reduction in cleaved caspase-1 level (Fig. 3). The lack of differential responses upon MCL-1 overexpression is not attributable to low transfection efficiency, as MCL-1 protein and mRNA levels were substantially elevated in transfected Raw 264.7 cells, as confirmed by western blot and qRT-PCR, respectively (Fig. S2A and S2B).

### MCL-1 does not inhibit pyroptosis triggered by *V. vulnificus* and *S.* Typhimurium infections

**Fig. 4.**
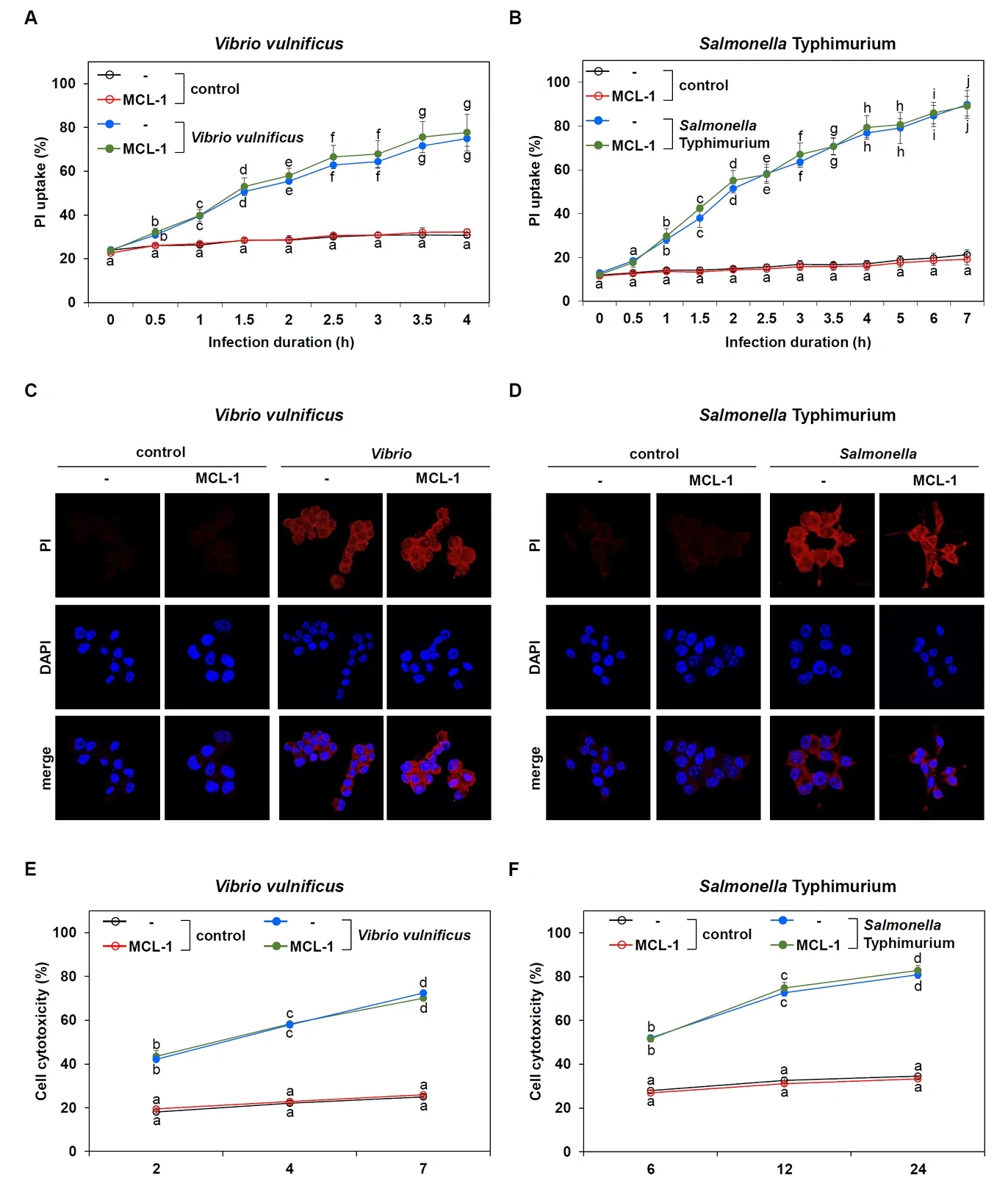
Lack of MCL-1 effect on pyroptosis induced by *Vibrio* and *Salmonella*. (A, B) Time-course analysis of PI uptake in Raw 264.7 cells transfected with control or MCL-1 expression vectors and infected with *V. vulnificus* M06-24/O for 30 min at an MOI of 20 (A) or *S.* Typhimurium ATCC 14028 at an MOI of 100 followed by incubation in DMEM containing 50 mg/ml gentamicin. Results are from three independent experiments performed in triplicates. Different letters denote statistically significant differences (*p* < 0.05). (C, D) PI staining was used to evaluate changes in cell membrane permeability in control (- : transfection with vector control) and MCL-1-overexpressing (MCL-1) Raw 264.7 cells after infection with *Vibrio* for 30 min at an MOI of 20 followed by incubation in DMEM containing 50 mg/ml gentamicin for 2 h (C) or *Salmonella* for 1 h at an MOI of 100 followed by incubation in DMEM containing 50 mg/ml gentamicin for 6 h (D). (E, F) LDH assays were performed to measure cytotoxicity in control (-) or MCL-1-overexpressing (MCL-1) Raw 264.7 cells infected with *V. vulnificus* for 30 min at an MOI of 20 (E) or *S.* Typhimurium for 1 h at an MOI of 100 (F) followed by incubation in DMEM containing 50 mg/ml gentamicin over the indicated time points. Results are from three independent experiments performed in triplicates. Different letters denote statistically significant differences (*p* < 0.05).

To further validate the MCL-1 involvement in pyroptosis, we assessed multiple indicators of pyroptotic cell death. Based on the optimized infection conditions, we monitored PI uptake over time as a measure of plasma membrane integrity—a hallmark of pyroptosis. PI uptake steadily increased in infected cells, but there was no significant difference between control and MCL-1-overexpressing cells for either *Vibrio* (Fig. 4A) or *Salmonella* (Fig. 4B) infections. Microscopic analysis of PI-stained Raw 264.7 cells confirmed these findings. Both infections led to prominent PI uptake in infected cells compared to controls, but no observable difference was detected between vector- and MCL-1-transfected cells (Fig. 4C and 4D).

To complement these findings, we performed lactate dehydogenase (LDH) release assays to quantify cell membrane damage associated with pyroptosis. LDH levels increased over time following bacterial infection, consistent with pyroptotic cell death (Fig. 4E and 4F). However, similar to the PI assay, no significant differences in LDH release were observed between control and MCL-1-overexpressing cells.

Thus, these results indicate that pyroptosis-evidenced by Gbp2 upregulation, caspase-11 activation, plasma membrane rupture, and cell lysis is not inhibited by MCL-1. Although MCL-1 showed marginal reduction of caspase-1 activation triggered by infections, this inhibition was insufficient to block pyroptosis. Collectively, pyroptosis induced by *V. vulnificus* and *S.* Typhimurium occurs independently of MCL-1 expression.

## Discussion

This study provides new insights into the molecular mechanisms underlying pyroptosis induced by *Vibrio vulnificus* and *Salmonella* Typhimurium infections, with a particular focus on the roles of Gbp2, the pro-apoptotic proteins Bak and Bax, and the anti-apoptotic protein MCL-1. While pyroptosis is increasingly recognized as a key innate immune defense mechanism against bacterial pathogens, the role of BCL-2 family proteins in modulating pyroptotic signaling remains largely unexplored. Our findings reveal an intricate interplay between canonical and non-canonical pyroptotic pathways and uncover distinct regulatory functions of Bak, Bax, and MCL-1 in this context.

We demonstrate that both *V. vulnificus* and *S.* Typhimurium robustly induce pyroptosis in murine cells through upregulation of Gbp2 and activation of caspases-1 and caspase-11. Notably, *S.* Typhimurium infection exhibited delayed pyroptotic kinetics compared to *V. vulnificus*, likely due to differences in intracellular trafficking dynamics or the timing of cytosolic LPS exposure. In MEF cells, both *Vibrio* and *Salmonella* infections triggered caspase-11 activation accompanied by Gbp2 upregulation, while caspase-1 remained inactive indicating that non-canonical pyroptosis is predominant in these fibroblasts (Fig. 1). In contrast, in macrophages, infection by either pathogen led to activation of both caspase-1 and caspase-11, along with strong Gbp2 induction (Fig. 3), confirming the involvement of both canonical (inflammasome-dependent) and non-canonical (inflammasome-independent) pyroptotic pathways. This dual activation underscores the complexity of host responses to intracellular Gram-negative bacteria and highlights the central role of Gbp2 in linking innate immune signaling and inflammasome activation (Kirkby et al., 2023; Pilla et al., 2014). Consistent Gbp2 induction across different cell types and pathogens further supports its critical function in regulating pyroptosis.

A key finding of this study is that Bak and Bax significantly influence pyroptosis, particularly through modulating Gbp2 expression and caspase-11 activation. In *V. vulnificus*-infected MEFs, both Gbp2 upregulation and caspase-11 activation were markedly impaired in *Bak*^-/-^ and *Bak*^-/-^*Bax*^-/-^ cells, with *Bax*^-/-^ cells showing a milder phenotype (Figs. 1A, 1B, and 2A). A similar, though less pronounced, pattern was observed in response to *S.* Typhimurium (Figs. 1C, 1D, and 2B). These results indicate that Bak-and to a lesser extent Bax-are required for optimal induction of the Gbp2-caspase-11 axis. Although the exact mechanism remains unclear, it may involve mitochondrial stress signaling that promotes Gbp2 expression or facilitates the release of mitochondrial-derived signals that enhance inflammasome activation. To our knowledge, this is the first study to demonstrate a critical role for Bak and Bax in these infections-induced pyroptosis, expanding their known functions beyond mitochondrial apoptosis.

Another significant aspect of this study is the investigation into the role of MCL-1, an anti-apoptotic member of the BCL-2 family, previously shown to inhibit apoptosis through interaction with Bak and Bax (Luo et al., 2021; Vogler et al., 2025). Unexpectedly, MCL-1 overexpression had no detectable effect on any pyroptotic markers examined-including Gbp2 expression, caspases activation, PI uptake, or LDH release-during *Vibrio* or *Salmonella* infections in macrophages (Figs. 3 and 4). These findings indicate that MCL-1 does not significantly regulate bacterial pyroptosis in this context, despite its established role in inhibiting Bak/Bax-dependent apoptosis. Interestingly, a recent study implicated the MCL-1–Bak axis in pyroptosis triggered by Rift Valley fever virus infection (Guan et al., 2024). Therefore, the involvement of BCL-2 family proteins in pyroptosis may be pathogen-specific and context-dependent.

Pyroptosis and apoptosis are distinct forms of cell death, yet recent studies have revealed regulatory cross-talk between them, particularly involving BCL-2 family proteins, which are central regulators of apoptosis. The BH3 domain, a conserved and essential motif within BCL-2 family members, mediates their interactions and functions (Chittenden, 2002). Interestingly, the pyroptotic effector GSDMD contains a BH3-like domain, allowing it to directly bind to anti-apoptotic BCL-2 proteins, such as BCL-2 itself. This interaction suppresses GSDMD cleavage and thereby limits pyroptosis (Shi and Kehrl, 2019). In addition, BAK activation resulted from MCL-1 downregulation triggers mitochondrial ROS (mtROS) production and release of oxidized mitochondrial DNA (mtDNA), which subsequently activates NLRP3 inflammasome and GSDMD-mediated pyroptosis during viral infection (Guan et al., 2025). These findings suggest that anti-apoptotic BCL-2 proteins may suppress pyroptosis both by directly binding GSDMD and by preserving mitochondrial integrity via inhibition of pro-apoptotic members BAK and BAX.

Maintaining mitochondrial membrane integrity restricts the cytosolic release of mtROS, a known upstream signal for the transcriptional activation of Gbp2 and for caspase-11 activation during pyroptotic cell death (Wang et al., 2019). In contrast, pro-apoptotic BAK and BAK permeabilize the outer mitochondrial membrane, allowing mtROS and mtDNA efflux into the cytosol (McArthur et al., 2018). This suggests a model in which BAK/BAX antagonize anti-apoptotic BCL-2 proteins, leading to MOMP and subsequent mtROS/mtDNA-mediated pyroptotic signaling, including Gbp2 induction and caspase-11 activation during bacterial infections. However, the exact molecular mechanisms underlying these interactions remain to be fully elucidated.

In conclusion, our study demonstrates that pyroptosis triggered by *V. vulnificus* and *S.* Typhimurium involves both canonical and non-canonical pathways and is positively regulated by the pro-apoptotic proteins Bak and Bax via Gbp2 and caspases 1/11. In contrast, MCL-1, despite its central role in apoptosis regulation, does not inhibit pyroptosis. These findings contribute to a broader understanding of the interplay between apoptosis and pyroptosis and uncover novel regulatory roles for BCL-2 family members in inflammatory cell death. Further studies are warranted to dissect the molecular mechanisms linking mitochondrial stress, Gbp2 regulation, and inflammasome activation in diverse cellular and pathogenic contexts.

## Supplementary Information

Fig. S1. Optimization of infection conditions in macrophage cells.

Fig. S2. Confirmation of MCL-1 ectopic expression.
