# Supplementary material for: Bak and Bax are crucial for Gbp2-mediated pyroptosis during Vibrio and Salmonella infections
Source: J Microbiol. 2025 Sep 30;63(9):e2508004. doi: 10.71150/jm.2508004 (PMC13577113; doi:10.71150/jm.2508004)
Supplement: Supplementary file 1 [file jm-2508004-Supplementary-Fig-S1.pdf]

Supplementary materials

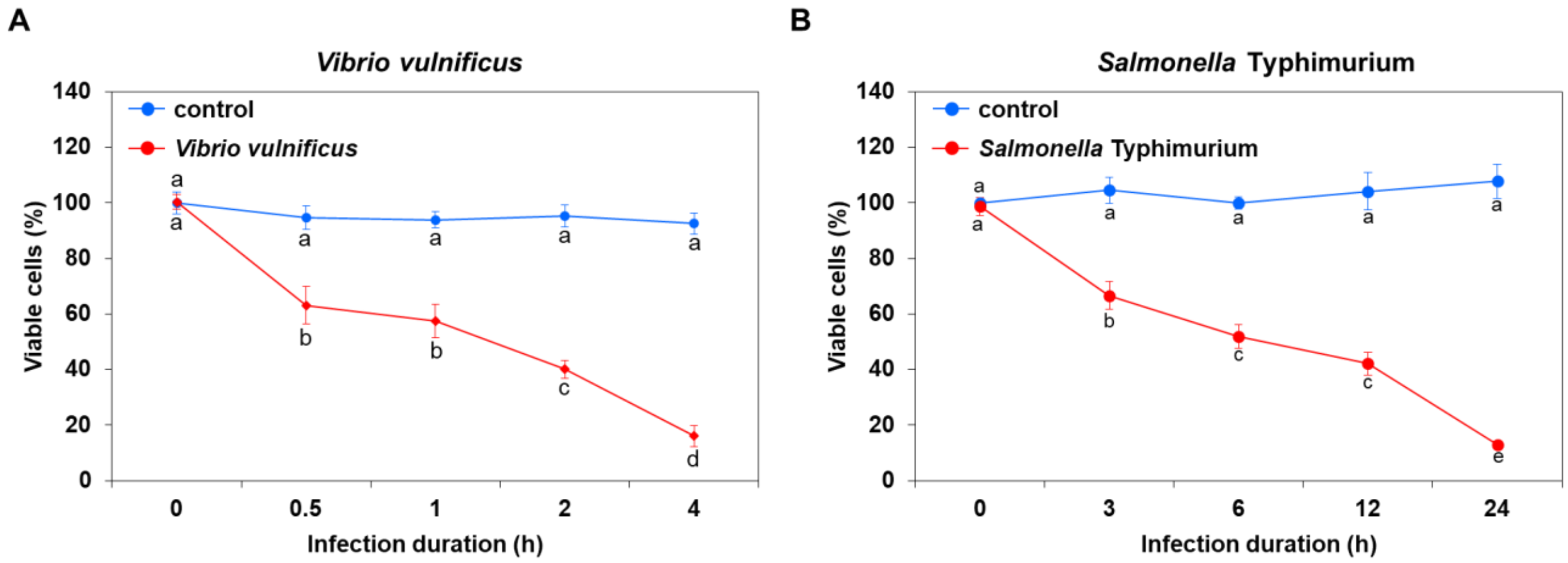

Fig. S1 Optimization of infection conditions in macrophage cells. Raw264.7 cells were infected with *V. vulnificus* M06-24/O (A) at a MOI of 20 for 30 min, or with *S.* typhimurium ATCC 14028 (B) at a MOI of 100 for 1 hour. After infections, cells were washed with PBS and incubated in DMEM containing 50 µg/mL gentamicin for the indicated durations. Cell viability was assessed using the trypan blue exclusion assay and expressed as the percentage of viable cells relative to the total. Results represent the mean ± SD of three independent experiments, each performed in triplicate. Different letters indicate statistically significant differences ($p < 0.05$).
