# Supplementary material for: Bak and Bax are crucial for Gbp2-mediated pyroptosis during Vibrio and Salmonella infections
Source: J Microbiol. 2025 Sep 30;63(9):e2508004. doi: 10.71150/jm.2508004 (PMC13577113; doi:10.71150/jm.2508004)
Supplement: Supplementary file 2 [file jm-2508004-Supplementary-Fig-S2.pdf]

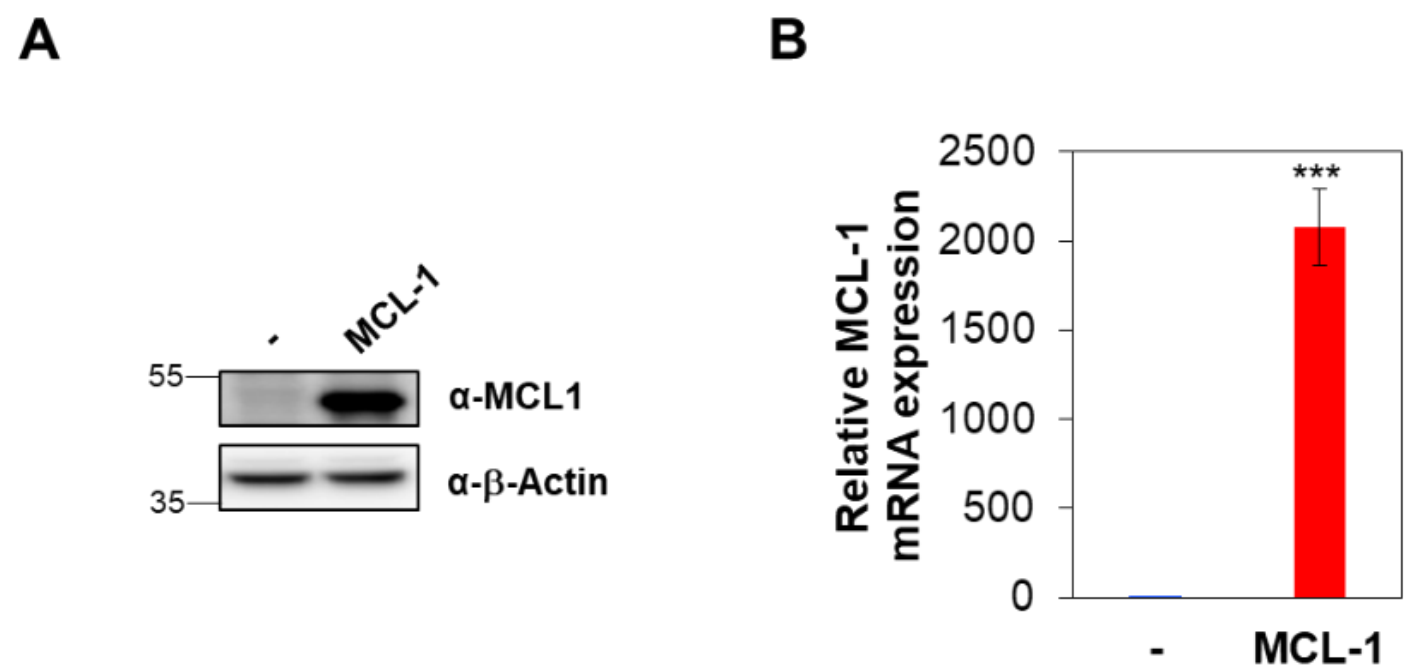

Fig. S2. Confirmation of MCL-1 ectopic expression. Levels of MCL-1 protein (A) and *MCL-1* mRNA (B) were detected by western blot and qRT-PCR, respectively, in MCL-1 transfected Raw 264.7 cells at 24 h post-transfection. Representative immune blot is shown (A). Data represent mean ± SEM from three independent experiments (***$p < 0.05$) (B).
